# Hyperspectral Technologies for Assessing Seed Germination and Trifloxysulfuron-methyl Response in *Amaranthus palmeri* (Palmer Amaranth)

**DOI:** 10.3389/fpls.2017.00474

**Published:** 2017-04-03

**Authors:** Maor Matzrafi, Ittai Herrmann, Christian Nansen, Tom Kliper, Yotam Zait, Timea Ignat, Dana Siso, Baruch Rubin, Arnon Karnieli, Hanan Eizenberg

**Affiliations:** ^1^The Robert H. Smith Institute of Plant Sciences and Genetics in Agriculture, The Robert H. Smith Faculty of Agriculture, Food and Environment, The Hebrew University of JerusalemRehovot, Israel; ^2^The Remote Sensing Laboratory, Blaustein Institutes for Desert Research, Ben-Gurion University of the NegevSede Boker Campus, Israel; ^3^Department of Entomology and Nematology, University of California, Davis, DavisCA, USA; ^4^State Key Laboratory Breeding Base for Zhejiang Sustainable Pest and Disease Control, Zhejiang Academy of Agricultural SciencesHangzhou, China; ^5^Institute of Agricultural Engineering, Volcani Center, Agricultural Research OrganizationBet Dagan, Israel; ^6^Department of Plant Pathology and Weed Research, Agricultural Research Organization, Newe Ya’ar Research CenterRamat Yishay, Israel

**Keywords:** herbicide resistance evolution, hyperspectral imaging and sensing, precision agriculture, proximal sensing, trifloxysulfuron-methyl

## Abstract

Weed infestations in agricultural systems constitute a serious challenge to agricultural sustainability and food security worldwide. *Amaranthus palmeri* S. Watson (Palmer amaranth) is one of the most noxious weeds causing significant yield reductions in various crops. The ability to estimate seed viability and herbicide susceptibility is a key factor in the development of a long-term management strategy, particularly since the misuse of herbicides is driving the evolution of herbicide response in various weed species. The limitations of most herbicide response studies are that they are conducted retrospectively and that they use *in vitro* destructive methods. Development of a non-destructive method for the prediction of herbicide response could vastly improve the efficacy of herbicide applications and potentially delay the evolution of herbicide resistance. Here, we propose a toolbox based on hyperspectral technologies and data analyses aimed to predict *A. palmeri* seed germination and response to the herbicide trifloxysulfuron-methyl. Complementary measurement of leaf physiological parameters, namely, photosynthetic rate, stomatal conductence and photosystem II efficiency, was performed to support the spectral analysis. Plant response to the herbicide was compared to image analysis estimates using mean gray value and area fraction variables. Hyperspectral reflectance profiles were used to determine seed germination and to classify herbicide response through examination of plant leaves. Using hyperspectral data, we have successfully distinguished between germinating and non-germinating seeds, hyperspectral classification of seeds showed accuracy of 81.9 and 76.4%, respectively. Sensitive and resistant plants were identified with high degrees of accuracy (88.5 and 90.9%, respectively) from leaf hyperspectral reflectance profiles acquired prior to herbicide application. A correlation between leaf physiological parameters and herbicide response (sensitivity/resistance) was also demonstrated. We demonstrated that hyperspectral reflectance analyses can provide reliable information about seed germination and levels of susceptibility in *A. palmeri*. The use of reflectance-based analyses can help to better understand the invasiveness of *A. palmeri*, and thus facilitate the development of targeted control methods. It also has enormous potential for impacting environmental management in that it can be used to prevent ineffective herbicide applications. It also has potential for use in mapping tempo-spatial population dynamics in agro-ecological landscapes.

## Introduction

In agricultural systems, weeds are the most important biotic factor and are responsible for more than 34% of crop yield losses worldwide (Oerke, 2006), thereby constituting a serious global challenge to agricultural sustainability and food security. The noxious weed *Amaranthus palmeri* S. Watson (Palmer amaranth) is one of the economically most important weeds, affecting commodity crops, such as cotton (*Gossypium* spp.), maize (*Zea mays* L.), and soybean (*Glycine max*) (Oliver and Press, 1994; Rubin, 2000; Massinga et al., 2001). More than that, this weed is also a problem in fields of less competitive, prostrate crops, such as, watermelon (*Citrullus lanatus*) and chickpea (*Cicer arietinum*) (Rubin and Matzrafi, 2015). In view of its high seed fecundity (Keeley et al., 1987), wide range of germination temperatures (Steckel et al., 2008), and C4 photosynthetic apparatus (Wang et al., 1992), *A. palmeri* may be regarded as a “super weed” (Guttmann-Bond, 2014).

Herbicides are considered as the most efficacious and cost-effective method for weed management. In the past, *A. palmeri* has been controlled mainly with three different classes of herbicide, acetolactate synthase (ALS) inhibitors, photosystem II (PSII) inhibitors, and 4-hydroxyphenylpyruvate dioxygenase (HPPD) inhibitors (Ward et al., 2013), but optimal management strategies are yet to be developed and concerns about the evolution of herbicide resistance remain to be addressed. This paper thus focuses on two key factors in the development of a sustainable long-term weed-management strategy, namely, estimating of the population of germinating seeds and evaluating herbicide susceptibility and resistance, and offers, for the first time, a non-destructive toolbox based on hyperspectral technologies and data analyses for the prediction of seed germination and herbicide response.

Fitness characters, such as seed germination, can have a significant effect on the robustness of the infesting field population and, as a consequence, on crop yield (Awan and Chauhan, 2016; Edelfeldt et al., 2016). This effect is predicted to be more extreme in the case of an aggressive noxious weed such as *A. palmeri* (Massinga et al., 2001; Ruf-Pachta et al., 2013). A negative correlation has been found between the viability of *A. palmeri* seeds and the depths to which the seeds are buried. Sosnoskie et al. (2013) showed that the deeper the burial depth, the lower germination rate. Seed dormancy can also inhibit seed germination, as has been demonstrated in a different species of *Amaranthus*, the common waterhemp [*A. tuberculatus* (Moq) Sauer]. Common waterhemp exhibits strong primary dormancy, which may be broken within 4 months after the ripening process, depending on the dormancy level (Wu and Owen, 2015).

Over the years, the intensive use herbicides have resulted in a strong selection pressure that has led to the evolution of herbicide-resistant weeds (Rubin, 1991). Resistance to several types of herbicide, including ALS, PSII and HPPD inhibitors, have been reported for *A. palmeri* (Ward et al., 2013). In particular, recent changes in herbicide regulations in Europe have led to increased use of ALS inhibitors (Kudsk et al., 2013), which is exacerbating concerns about the evolution of ALS resistance in *A. palmeri* populations and other weeds (Sibony and Rubin, 2003; Délye et al., 2011; Nandula et al., 2012; Matzrafi et al., 2015). One of the problems in monitoring the development of herbicide resistance is that it is usually conducted retrospectively using *in vitro* destructive molecular (Délye et al., 2015), physiological (Dinelli et al., 2008; Godar et al., 2015; Kleinman et al., 2015) and/or biochemical (Edwards and Cole, 1996; Tal et al., 1996; Matzrafi et al., 2014) methods. The weed science community has therefore recognized the need for methods to detect herbicide resistance at early stages of weed emergence before the herbicide is applied (Délye et al., 2015).

A possible means to facilitate the early detection of weeds lies in hyperspectral technologies. Such technologies are already in wide use in agriculture for such diverse applications as: (1) predicting seed germination (Nansen et al., 2015); (2) distinguishing between pest-infested and non-infested seeds (Nansen et al., 2014); (3) monitoring crop responses to biotic stressors (Prabhakar et al., 2012; Nansen and Elliott, 2016); (4) assessing the leaf area index (LAI) of wheat (*Triticum aestivum*) and potato (*Solanum tuberosum*) (Herrmann et al., 2011); and (5) determining – using near infrared (NIR) – rapeseed quality, i.e., seed weight and total oil content and oil fatty acid composition (Velasco et al., 1999). In addition, weed science studies have used hyperspectral methods to distinguish between weeds and crops (Okamoto et al., 2007; López-Granados et al., 2008; Herrmann et al., 2013). The use of reflectance-based analyses can therefore be exploited to prevent ineffective or needless applications of herbicides, slow down the evolution of herbicide resistance and to map the distribution (and the possible spread) of resistant *A. palmeri* populations in agro-ecological landscapes. To the best of our knowledge this is the first study exploring a method implementing hyperspectral means in order to estimate *A. palmeri* infestation and herbicide response.

In the current study, hyperspectral methods form the basis of a method that facilitates the use of *ex situ* and *in vivo* non-destructive methods for estimating seed germination and herbicide response, respectively. With the ultimate aim to better understand the invasiveness of *A. palmeri* and hence facilitate the development of more targeted control methods, the current study addressed four specific aims: (***i***) to examine the accuracy and utility of hyperspectral imaging to predict the germination of *A. palmeri* seeds; (***ii***) to investigate the extent to which hyperspectral reflectance data from *in vivo* leaves of young *A. palmeri* can be used to detect and assess their response to the ALS inhibitor, trifloxysulfuron-methyl; (***iii***) to spectrally assess physiological parameters prior to herbicide application; and (***iv***) to explore image processing as a tool for evaluating herbicide response. The current study is also aiming to show feasibility for spectral assessment of weed response prior to herbicides application. Ability to estimate response to herbicide will be a game changer in the field of weed management and will allow early identification of resistant weeds creating better and efficient weed management.

## Materials and Methods

### Plant Material and Herbicide Treatment

Three *A. palmeri* seed populations were collected from two corn fields (designated NA1 and NA2) at Kibbutz Na’an, Israel (31°53′01″N 34°51′28″E) and from a cotton field (designated BM1) at Moshav B’ney Darom, Israel (31°49′11″N 34°41′30″E). These fields were selected for two reasons: a long history of the use of herbicides, including ALS inhibitors (trifloxysulfuron-methyl and pyrithiobac-sodium), and recent reports of herbicide resistance by the farmers. Mature seeds from 30 *A. palmeri* plants were collected in each field, and the collected seeds from each field were considered as one “population.” The seeds were air dried and stored at 4°C for at least 2 months before being used in this study. A total of 120 seeds (40 from each population) were imaged individually and subsequently tested for germination, as follows. Seeds were sown into pots (7 cm × 7 cm × 6 cm) containing 100% growth mixture, constrained of tuff, coconut and kavul in varying ratios (Tuff Marom-Golan, Ram 6, Israel) and left to germinated in a net house under summer conditions (30–35°C). Germination was assessed 7–10 days after sowing (DAS) (Guo and Al-Khatib, 2003), and seed viability was recorded as “germinated” or “non-germinated” seeds.

From the original 120 seeds, we obtained 67 plants (germinated seeds), which were subsequently used in studies of susceptibility to trifloxysulfuron-methyl (Envok, 75% SL, Syngenta, Basel, Switzerland). Twenty-one days after emergence (DAE), when the plants had three to four true leaves (after leaf gas-exchange and hyperspectral leaf data measurements had been obtained; see below), individual *A. palmeri* plants were treated with trifloxysulfuron-methyl at the equivalent rate of 11.25 g ai. h^-1^ mixed with 0.15% of the surfactant alkylaryl polyether alcohol (DX spreader, 800 g ai L^-1^, Agan Chemical Manufacturers Ltd., Ashdod, Israel). Trifloxysulfuron-methyl was applied using a chain-driven sprayer delivering 300 L ha^-1^. The experiment was arranged in a completely randomized factorial design inside a net house under summer conditions (25/35°C, night/day). To determine the plant response to trifloxysulfuron-methyl, fresh shoot biomass was recorded 21 days after treatment (DAT), i.e., at 42 DAE. Plants were initially grouped according to their visual injury, taking under consideration of their survival odds under crop-weed competition conditions. Five plants of each seed population served as the untreated control (without herbicide application).

### Leaf Gas-Exchange Measurements

At 21 DAE, leaf gas-exchange measurements were conducted with a Li-6400 portable photosynthesis and fluorescence measurement system (6400-40 leaf-chamber fluorometer; Li-Cor, Inc., Lincoln, NE, USA). All 67 plants were measured for: predicted photosynthetic rate, stomatal conductance and PSII efficiency. The measuring chamber enclosed a circular 2-cm^2^ section of leaf area and calculated the gas flow on both sides of the leaf. The air flow rate was kept constant at 500 μmol m^-2^ s^-1^, and the reference CO_2_ concentration was 400 μmol CO_2_ mol^-1^ air (ppm). Light intensity was monitored prior to each measurement and kept constant at 1200 μmol photons m^-2^ s^-1^ (10% blue light). Leaf gas-exchange measurements were conducted in a net house during the day (9:00–11:00 am) under summer conditions: temperatures of 30–35°C, relative humidity of 45–55%, and radiation flux of 1000–1100 μmol m^-2^ s^-1^. To assure homogeneity, all leaf gas-exchange measurements were acquired from the third fully expanded leaf from the top of the plant. The rate of carbon assimilation (μmol CO_2_ m^-2^ s^-1^) and the rate of stomatal conductance of water vapor (mol H_2_O m^-2^s^-1^) were determined. Chlorophyll a fluorescence was assessed using equation 1: The quantum yield of PSII, ∅PS_2_, was calculated as follows.

(1) $$\begin{array}{c} \emptyset PS_{2}\;=\;\frac{F_{m}^{'}-F_{t}}{F_{m}^{'}} \end{array}$$

where *F*_t_ is the steady-state fluorescence, and $F_{m}^{\prime }$ is the maximal fluorescence in the light-adapted state.

### Hyperspectral Imaging of Seeds

A pushbroom hyperspectral camera (PIKA II, Resonon Inc., Bozeman, MT, USA) was mounted 40 cm above the seeds, and hyperspectral images were acquired under artificial light (two 15-W, 12-V light bulbs mounted on either side of the lens), with a spatial resolution of 50 pixels per 2 mm. The main specifications of the hyperspectral camera were: Firewire interface (IEEE 1394b), 12-bit digital output, 240 spectral bands from 392 to 889 nm (spectral resolution = 2.1 nm) by 640 pixels (spatial). The objective lens had a 35-mm focal length (maximum aperture of F1.4) with a 7° field of view, optimized for NIR and visible–NIR spectra. A piece of white Teflon (K-Mac Plastics, Grand Rapids, MI, USA) was used for white calibration. Relative reflectance with reference to the reflectance from white Teflon was determined. Colored plastic cards (green, yellow, and red) were imaged at all hyperspectral imaging events, and average reflectance profiles from these cards were used to confirm the high consistency of hyperspectral image acquisition conditions (less than 2% variance within individual spectral bands).

**FIGURE 1:**
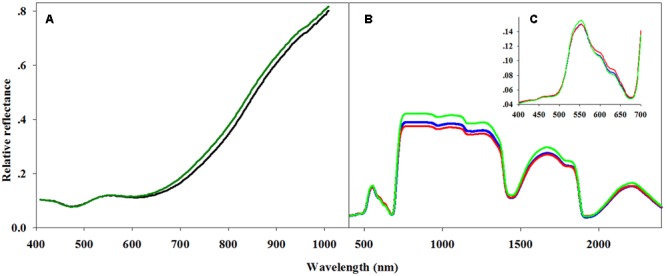
**Averaged spectra for hyperspectral analysis of seeds and leaves. (A)** Two seed reflectance classes: germinating (dark green line) and non-germinating seeds (black line), **(B)** three classes, resistant (green line), moderate (blue line) and sensitive (red line), of general leaf reflectance, and **(C)** with a focus on the 400–700 nm spectral region covering the visible spectrum.

The hyperspectral imaging data from the seeds was processed as following. All hyperspectral imaging files were converted into ASCII code and imported into the Statistical Analysis System (SAS) software package for processing and data classification. The first 14 and last 5 spectral bands were omitted from each hyperspectral data file, as these were considered to be associated with stochastic noise. Consequently, only 221 spectral bands from 423.6 to 878.9 nm were included in the analysis (**Figure 1A**).

For the hyperspectral imaging analysis of the seeds, 120 seeds were randomly divided into three equal groups, and each group was tested as an independent validation set. This procedure was repeated three times in order to build linear discriminant analysis (LDA; Fisher, 1936) classification models. The results of the three models were averaged.

### Acquisition and Analysis of Hyperspectral Leaf Reflectance Data

The hyperspectral leaf reflectance data were obtained from the adaxial side of the same leaf that had been measured earlier for gas exchange. Hyperspectral data were obtained using an Analytical Spectral Devices (ASD) spectrometer FieldSpec 4 high resolution (ASD Inc., Boulder, CO, USA) having a range of 350–2500 nm, with the optic fiber connected to a contact probe (ASD Inc.). The contact probe had a tungsten halogen light source. To obtain pure reflectance of the leaf alone, a black metal plate was placed underneath each leaf during the spectral measurement; all 67 leaves were covered in the entire field of view. The spectrometer was programmed to average 10 spectra for each sample measurement, and white reference measurements were performed several times throughout the experiment. The dark current was applied automatically by a shutter in the spectrometer in accordance with the optimization for the lighting conditions facing the white reference panel (Hatchell, 1999). The spectral output was given automatically as relative units with 1 nm intervals, where relative units were obtained by dividing each target measurement by the last acquired white reference measurement.

To facilitate analysis of the data, the edges of the spectral range that were assumed to be noisy were eliminated, and the range was set to 400–2400 nm. To assess the ability to spectrally predict photosynthetic rate, stomatal conductance and PSII efficiency, the partial least squares regression (PLS-R) method was applied. The PLS-R is a practical predictive tool for hyperspectral data (Hansen and Schjoerring, 2003; Herrmann et al., 2011) and it was chosen because it can deal efficiently with the multi-collinearity of the reflectance values of the hyperspectral data (Wold and Eriksson, 2001; Atzberger et al., 2010). A PLS-R model was constructed for each of the measured plant properties, namely, photosynthetic rate, stomatal conductance and PSII efficiency, and each model was cross-validated using the Venetian blinds method. Each model was assessed in terms of its coefficient of determination (*R*^2^), the root mean square error of calibration (RMSEC) and the RMSE of cross-validation (RMSECV).

To assess the ability to assign hyperspectral leaf data to different classes of herbicide response, PLS discriminant analysis (PLS-DA) was applied to allow maximal separation among the predefined classes (in this case, sensitive, moderate response, and resistant to the herbicide). This method has been used previously to differentiate between broadleaf weeds, grass weeds and wheat (Herrmann et al., 2013). To combine the PLS (numerical method) with the DA (categorical method), each class was assigned a binary artificial sequence of arbitrary numbers. This sequence was assigned to all the class samples; the size of the sequence was set by the number of classes (Musumarra et al., 2004; Xie et al., 2007). Spectral samples were used to build a PLS-DA model that was cross-validated using the Venetian blinds method. The cross validation results for the model are presented in the Results section. The classification quality was assessed by the accuracy figures presented in a confusion matrix and the matrix’s Cohen’s Kappa as presented and defined by Cohen (1960). The PLS-R and PLS-DA models and their post processing were run in a Matlab 7.6 (MathWorks, Natick, MA, USA) environment using the PLS toolbox (Eigenvector Research Inc., Wenatchee, WA, USA).

### Herbicide Response Bulk Analysis of Different *A. palmeri* Populations

To determine whether the plants could be bulked instead of being analyzed as three different populations, two different statistical analyses (Tukey–Kramer and a leave-one-out cross-validation) were performed. The Tukey–Kramer test was performed using JMP Pro 12 (SAS Institute Inc., Cary, NC, USA). In the leave-one-out analysis, the average was calculated three times with different population set aside each time (Equation 2).

(2) $$\begin{array}{c} \bar{X}\;=\;\frac{X_{1}\times n_{1}\;+\;X_{2}\times n_{2}}{\left(n_{\mathrm{total}}\;-\;n_{3}\right)} \end{array}$$

where $\bar{X}$ is the one-population-out average, *X*_i_ is the population average, *n*_i_ is the number of plants in the population, and *i* describes the tested population. According to both the Tukey–Kramer test and the cross-validation test, there were no significant differences between the three populations (Supplementary Table S1).

### Assessing Plant Response to Trifloxysulfuron-methyl Using Digital Imaging Technology

To assess plant response to trifloxysulfuron-methyl, all plants (treated and untreated) were photographed 21 DAT. Photographs were taken with an off-the-shelf digital camera (Canon, PowerShot SX20 IS^®^) placed on a tripod, positioned at a 45° angle from the pot, at a distance of 1.2 m and against a black background. The 8-bit JPEG images analysis and processing were performed using Matlab and the public-domain software ImageJ (NIH)^1^. To assess plant weight based on the images, the first parameter to be analyzed was the mean gray value (MGV), and the thresholds were determined to include all green organs based on the hue, saturation and brightness (HSB) of an 8-bit JPEG image. The MGV was calculated from the average gray scale value of the pixels in the selected area for the HSB threshold using Equation 3.

(3) $$\begin{array}{c} grayvalue=0.299R+0.587G+0.114B \end{array}$$

where R, G, and B stand for the three spectral regions: red, green, and blue, respectively. In all the 8-bit JPEG images, the setting of the different color components in each pixel was determined on the basis of the R, G, and B 8-bit (2^8^) intensity graduations values, ranging from 0 to 255.

The second parameter to be determined was the area fraction (AF), which was calculated in Matlab based on images from all plants. The thresholds were determined to include all shoot tissue pixels based only on the brightness channel. AF was calculated as the sum of all of the pixels in the selected area (*SA*) divided by the total number of pixels in the image (*totA*; Equation 4).

(4) $$\begin{array}{c} AF\;=\;\frac{SA}{totA} \end{array}$$

Data obtained from the photographs and data of shoot biomass (fresh weight, FW) were analyzed to determine the correlation between plant weight and the AF or MGV values. The data were analyzed using SigmaPlot software (ver. 10, Systat Software Inc., San Jose, CA, USA). A non-linear regression model [polynomial, linear (Equation 5)] was developed to analyze the correlation of the recorded weights from the different plants with the different AF and MGV values.

(5) $$\begin{array}{c} {f\;=\;y}_{0}+ a*x \end{array}$$

where *y*_0_ – the value of AF or MGV measured with ImageJ, *a* – the slope of the curve and *x* – the shoot FW (% of control).

### DNA Extraction and Molecular Studies to Detect Target Site Resistance to ALS Inhibitors

Mutations in the *ALS* gene can endow herbicide resistance due to structural modifications in the herbicidal target site (Tranel et al., 2016). To detect structural substitutions, the *ALS* gene was sequenced and analyzed. A section of leaf tissue (3 cm^2^) was excised from each treated plant. Each leaf section was placed in its own microtube. DNA was extracted using the Puregene DNA isolation kit (Gentra Systems, Minneapolis, MN, USA) according to the manufacturer’s instructions and diluted 10-fold before further use. Primers were used to identify the gene and locate the common point mutations that can endow altered target sites. Known primers were used to sequence the *ALS* gene from *A. palmeri* (Sibony and Rubin, 2003; Manor, 2011).

All polymerase chain reaction (PCR) amplifications were performed in 25 μL with a final concentration of 0.20 μM of each dNTP and 0.25 μM of each primer. The cycling program began with 4 min at 94°C, followed by 37 cycles, each consisting of 30 s at 94°C, 30 s at 57°C and 30 s at 72°C. The program ended with a final step of 4 min at 72°C. PCR products were separated on agarose gels (1.5%) to confirm the amplicon size, and each strand was sequenced using the same specific primers (Supplementary Table S2). Sequence analyses and alignment were performed using the BioEdit software (Hall, 1999). The obtained sequences were compared to known sequences of the *ALS* genes from *Arabidopsis thaliana* (X51514).

## Results

### Hyperspectral Seed Imaging for Germination Test

Seed germination was recorded 7–10 DAS (Supplementary Table S3) and data were correlated with data from reflectance measurements. Based on the LDA classification method, 67 seeds were identified as germinating and 53 as non-germinating, with accuracy (the ability to correctly identify each class) rates of 81.2% for the identification of germinated seeds and 77.3% for the identification of non-germinated seeds (**Table 1**). **Table 1** presents the distribution of the seed samples in terms of germination success among the three populations. The accuracies are of the ability to correctly identify each of the two classes.

**Table 1:** Seed distribution according to populations and germination model validation results.

Population	Germinating	Non-germinating	Total
NA1 (# of samples)	14	26	40
NA2 (# of samples)	30	10	40
BM1 (# of samples)	23	17	40
Total (# of samples)	67	53	120
Accuracy	81.21%	77.3%	
Standard deviation of accuracy	2.56	2.64	

*n = 120; Kappa = 0.58.*

### Grouping the *A. palmeri* Plants according to Their Response to Trifloxysulfuron-methyl

Individual plants were grouped according to their response to trifloxysulfuron-methyl, as sensitive, moderate response, or resistant, according to whether they accumulated 0–20%, >20 to ≤40%, or >40%, respectively, of the biomass of the untreated control (Supplementary Table S3). The method of dividing the plants into different groups according to their response to the herbicide was examined and validated through the use of a chi-square test (*P* > 0.75). Out of the 67 plants used in the study, 13 plants were classified as resistant, 30 as moderate response, and 24 as sensitive (**Table 2**). This grouping method also reduces the effect contributed by the initial genetic differences and highlights the effect of environmental factors on herbicide response.

**Table 2:** Distribution of *A. palmeri* population response groups under trifloxysulfuron-methyl treatment.

		Resistant	Moderate	Sensitive	Total count (% of total)
NA1	Count	4	5	5	14
	Total %	5.97	7.46	7.46	20.90
	Col %	30.77	16.67	20.83	
	Row %	28.57	35.71	35.71	
NA2	Count	4	14	12	30
	Total %	5.97	20.90	17.91	44.78
	Col %	30.77	46.67	50.00	
	Row %	13.33	46.67	40.00	
BM1	Count	5	11	7	23
	Total %	7.46	16.42	10.45	34.33
	Col %	38.46	36.67	29.17	
	Row %	21.74	47.83	30.43	
Total Count		13	30	24	67
Total %		19.40	44.78	35.82	

*n = 67; (*Prob* > 0.7514).*

To eliminate the possibility of a target site resistance mechanism, we sequenced the *ALS* genes of 5–10 individuals from each response group. No alteration of the *ALS* gene that could be associated with target site resistance was found (Supplementary Figure S1).

### Determination of the Response of *A. palmeri* to Trifloxysulfuron-methyl Using Hyperspectral Leaf Data

Using PLS-DA, we created a classification model that distinguishes between the three classes of herbicide response (sensitive, moderate response, resistant) based on the full spectral range (400–2400 nm; **Figure 1B**). The attempt to distinguish between the three classes based on cross-validation had a total accuracy of 50.7% (**Table 3**). For distinguishing solely between sensitive and resistant individuals (i.e., two classes), the total accuracy increased to 86.5% (**Table 4**).

**Table 3:** Confusion matrix for distingushing between three response groups (resistant response, moderate response and sensitive response).

	Resistant	Moderate	Sensitive	Total predicted as	User accuracy (% correct)
Resistant	8	11	2	21	38.1
Moderate	4	7	3	14	50
Sensitive	1	12	19	32	59.4
Total actual class	13	30	24	67	
Producer accuracy (% correct)	61.5	23.3	79.2		50.7

*Confusion matrix for the 400–2400 nm spectral range in the PLS-DA cross-validation model. *n* = 67, Kappa = 0.27.*

**Table 4:** Confusion matrix for distingushing between two classes (resistant and sensitive).

	Resistant	Sensitive	Total predicted as	User accuracy (% correct)
**Full spectral range 400–2400 nm**				
Resistant	11	3	14	78.6
Sensitive	2	21	23	91.3
Total actual class	13	24	37	
Accuracy (% correct)	84.6	87.5		86.5
**Visible spectral range 400–700 nm**				
Resistant	10	1	11	90.9
Sensitive	3	23	26	88.5
Total actual class	13	24	37	
Accuracy (% correct)	76.9	95.8		89.2

*Confusion matrix for the 400–2400 nm and 400–700 nm spectral ranges in the PLS-DA cross-validation model. 400–2400 nm (*n* = 37, Kappa = 0.71); 400–700 nm *(n* = 37, Kappa = 0.75).*

Variable importance in projection (VIP) was used to explore the importance of the connection between spectral regions and the plants’ herbicide response (Supplementary Figure S2 and Table S4). VIP values show the importance of each wavelength to the model (Cohen et al., 2010). This method was applied for the two-class PLS-DA classification model as presented by Wold et al. (1993). The two-class VIP model (Supplementary Figure S2 and Table S5) shows the VIP values and their peaks at 400–700 and 1850–2000 nm (**Figures 1B,C**). Therefore, a PLS-DA classification model of the same two classes was applied for each individual spectral region. Examination of the cross-validation results from the visible spectral range showed a higher level of total accuracy: 89.2% (**Table 4**).

### Relationship between Physiological Characteristics and the Response of *A. palmeri* to Trifloxysulfuron-methyl

**FIGURE 2:**
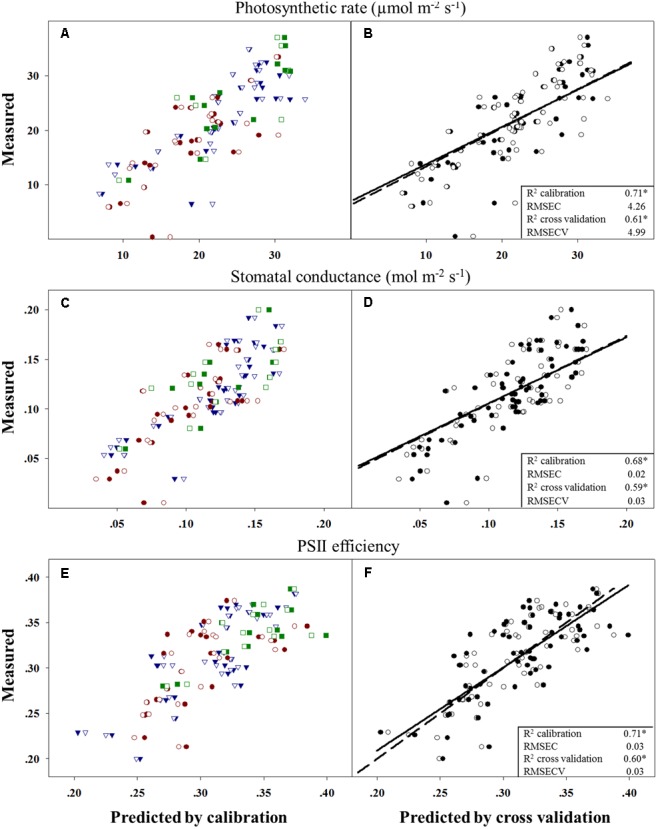
**Correlation between measured and predicted values in the calibration to (filled shapes/full line) and cross-validation (open shapes/dashed line) of all three physiological variables.** Measured vs. predicted values for all 67 plants divided into the three response groups and by prediction method: moderate response (blue), sensitive (red) and resistant (green) **(A,C,E)**. Data for parameter comparisons conducted using the two methods: **(B)** photosynthetic rate, **(D)** stomatal conductance and **(F)** PSII efficiency (PSIIE). *n* = 67; ^∗^*p* < 0.001.

Evolutionary changes contributing to herbicide resistance can be correlated with different adaptive traits. We tested the differences in three physiological variables – photosynthetic rate, stomatal conductance, and PSII efficiency – in corelation with plants’ response to the herbicide. Calibration and cross-validation data sets were fitted against measured data to determine the correlation between different herbicide response groups and data sets (**Figures 2B,D,F**). The obtained *R*^2^ values for the calibration and cross-validation analyses were 0.71 vs. 0.61 for photosynthetic rate, 0.68 vs. 0.59 for stomatal conductance, and 0.71 vs. 0.60 for PSII efficiency (**Figures 2A,C,E**). The strong significant correlation between the measured and the predicted values indicates an actual relationship between these productivity traits and herbicide response (**Figures 2A,C,E** and **Table 5**). Herbicide response was found to correlated with higher physiological capacities. The resistant plant group exhibited significantly higher (*p* ≤ 0.05) mean values for all three productivity traits, as compared to the sensitive group: 27.6 vs. 17.66 for photosynthetic rate, 0.14 vs. 0.1 for stomatal conductance, and 0.34 vs. 0.28 for PSII efficiency (Supplementary Table S6).

**Table 5:** Output of PLS-R cross-validation models for photosynthetic rate, stomatal conductance and photosystem II efficiency.

Model name	Photosynthetic rate	Stomatal conductance	Photosystem II efficiency
*R*^2^ calibration	0.709^∗^	0.684^∗^	0.707^∗^
RMSEC	4.26	0.023	0.027
Latent variable	6	6	6
*R*^2^ cross-validation	0.610^∗^	0.590^∗^	0.595^∗^
RMSECV	4.99	0.027	0.032

*n = 67; *^∗^p* < 0.001.*

### Response of *A. palmeri* Plants to Trifloxysulfuron-methyl Assessed Using Imaging Technology

**FIGURE 3:**
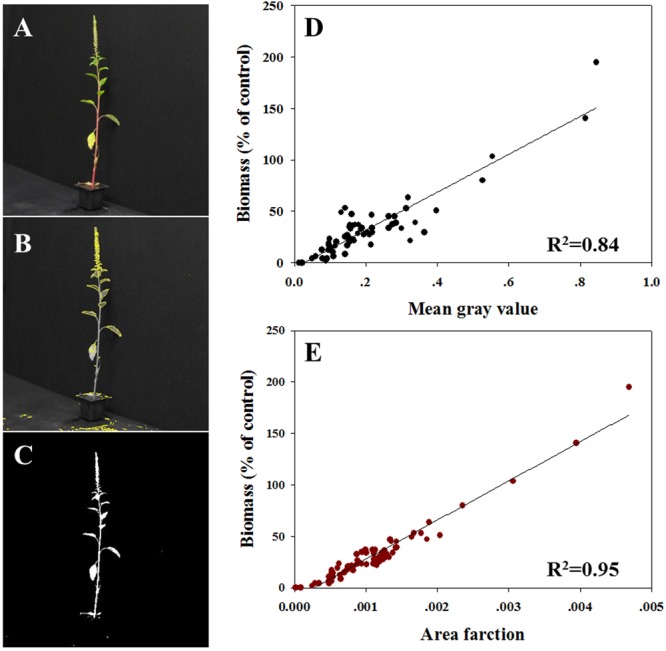
**Correlation between biomass (% of control) to two different parameters measured using ImageJ software. (A)** Example of an actual digital photograph of a plant; **(B)** example of a picture of the selected area used for MGV assessment; **(C)** example of a picture of the selected area used for AF analysis; **(D)** MGV per plant vs. biomass, and **(E)** AF where all pixels per plant vs. biomass.

All plants were photographed digitally (**Figure 3A**) to allow area measurement of MGV (**Figure 3B**) and AF (**Figure 3C**). So as to refer only to the productive traits of the plant (defined by the green tissue), the thresholds based on HSB values were adjusted in the pictures of surviving plants. Initial variables for the ImageJ software were: hue: 45–115; saturation: 22–255; and brightness: 68–255. MGV and AF were determined using the macro record for the threshold area and batch-processing for the rest of the images. MVG was found to be highly correlated with the measured biomass (*R*^2^ = 0.84; **Figure 3D**). So as to refer to the entire plant shoot, the brightness channel was used in an adjusted range of 0.50–1 (equivalent to 128–255 nm). The data analysis revealed a strong correlation between AF measured under these conditions and measured biomass (*R*^2^= 0.95; **Figure 3E**). Data shown here indicates that the AF parameter is more suitable for the prediction of absolute plant biomass, whereas plant survival and health are better predicted with the MGV.

### Description of Germination Prediction and Herbicide Control of *A. palmeri*

**FIGURE 4:**
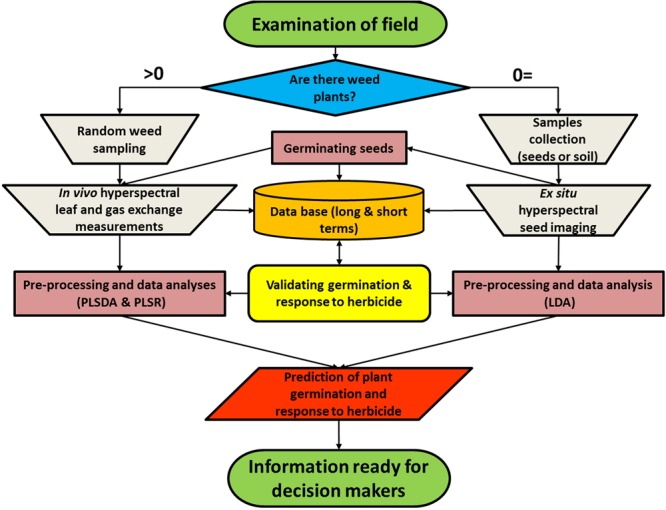
**A bi-model showing the process of data collection from a specific weed population to produce predictions of plant germination and herbicide response**.

We propose a bi-model (**Figure 4**) that uses reflectance data from seed imaging and hyperspectral data for leaves together with leaf physiological characteristics to predict both germination and herbicide response in a weed population. The first step is to obtain samples for spectral measurements: if there are weeds growing in the field, they are spectrally measured, and seed samples are collected for laboratory experiments. Seeds are cleaned of soil, imaged indoors, and transferred to soil-filled pots for germination in order to obtain germination validation data. Plants are then grown under controlled conditions for leaf hyperspectral measurements, followed by herbicide application for validation purposes. The analyses are based on validation of the germination obtained by germination tests as well as response to herbicide obtained by examination of plant biomass and survival rate at 21 DAT. In the current study, plants were grown in pots and measured in a net house, allowing validation of both germination and herbicide application. The outputs of the model enable both prediction of germination and response to herbicide.

## Discussion

In this study, we present novel, non-destructive methods for the estimation of seed germination and herbicide response in *A. palmeri* prior to herbicide application. At present, resistance is detected retrospectively (Steckel et al., 2008; Goggin et al., 2016; Rey-Caballero et al., 2016), and methods for the detection of herbicide resistance are based on time-consuming processes such as pre- or post-emergence herbicide application and heredity tests (Burgos et al., 2012). These methods result in at least one season of yield loss, often unnecessary multiple applications of herbicide, and long-term damage reflected in the enrichment of the seed bank with resistant seeds. Early detection of herbicide resistance may slow its evolution and can serve as a jumping-off point for developing alternative management practices to slow the spread of the phenomenon.

Seed hyperspectral imaging was found to be ∼80% accurate for germination prediction. An examination of the hyperspectral data obtained from leaves had 86.5% total accuracy for classification, based on two response groups (sensitive and resistant) instead of three (sensitive, intermediate, and resistant). When the spectral range was reduced to visible, the accuracy still remained relatively high, ∼89.2%. The resistant response group had higher mean values for all three physiological variables (photosynthetic rate, stomatal conductance, and PSII efficiency) than the sensitive group (Supplementary Table S6), which provided further support for the novel methodology presented in the current study. In most cases, due to its dominance, target site resistance divides the population into two phenotypic groups (sensitive and resistant). Sequencing individuals from all response groups, haven’t reveal any known substitutions associated with resistance to ALS inhibitors. Similar cases of non-target site resistance to ALS inhibitors have previously been reported, and there is evidence that the involvement of a single gene encoding for cytochrome P450 enzymes can endow this resistance (Yamada et al., 2000; Gion et al., 2014). This type of resistance mechanism can be correlated with the effect of one gene with two alleles, creating three levels of response to the herbicide (sensitive, moderate, and resistant). Plants’ response to trifloxysulfuron-methyl can also be endowed by other non-target site resistance mechanisms but our results eliminate the possibility of a target site resistance mechanism in our plants, reinforcing the validity of our three group analysis method. Further study is needed to better understand the correlation between herbicide response and different physiological traits. Hyperspectral analyses might be an efficient tool for achieving these goals.

In agriculture, hyperspectral techniques are already being applied to detect different traits in food products (ElMasry et al., 2007; Kamruzzaman et al., 2012; Ignat et al., 2014). Two particular studies describe work that impinges on our own: one reports the potential of NIR spectroscopy for the simultaneous analysis of seed weight, total oil content and oil fatty acid composition in intact single seeds of rapeseed (Velasco et al., 1999), and the other describes the use of NIR to discriminate between viable and empty seeds of *Pinus patula* Schiede and Deppe (Tigabu and Odén, 2003). In light of the above work, we hypothesized that the sequence of events leading up to germination and the accompanying changes in the contents of metabolites in the seeds would allow us to distinguish between *A. palmeri* seeds that are ready to germinate and a seeds that are not ready to germinate (dormant) or are non-viable. In the current study, we have shown a robust model (described below) for the detection of germination ability of *A. palmeri*.

In weed science, hyperspectral imaging has previously been used for site-specific weed management, particularly for weed crop classification (López-Granados, 2011; Herrmann et al., 2013; Shapira et al., 2013) or as a part of decision support system for herbicide application (Tellaeche et al., 2008; Lati et al., 2011). We could not find any reference to the use of this technology for early detection of herbicide response in young weeds (phenological stage of 3–4 true leaves). The model presented in the current study (**Figure 4**) merges seed and leaf assessment by hyperspectral technologies. Each of the model branches (i.e., seeds and plants) can be operated alone, but for optimal comprehensive weed management it is recommended that both branches of the model be applied. The output can be used by variety of decision makers. All the information acquired is entered into a database, which in the current era of big data will have a variety of immediate and potential uses: The database will find utility for applying data mining techniques for each run of the model as well as for long-term data collection and analyses that can also produce a spatial distribution of herbicide resistance. The imaging techniques described here can be used to predict seed germination, giving the farmer an indication of the following year’s field population and an evaluation of weed infestation in the field. Chemical weed control can be applied both pre- and post-weed emergence (Yadav et al., 2016); certain compounds, such as pendimethalin (tubulin interaction inhibition), can serve for both purposes (Riches et al., 1997; Yaduraju et al., 2000). The low benefit that is derived from pre-emergence herbicidal treatment is related largely to the uncertainty about the subsequent year’s weed infestation rates. The seed data presented here can be used in a pre-season decision support system determining whether herbicides should be applied pre- or post-emergence. The non-destructive hyperspectral leaf methodology can provide immediate results and recommendations for the current season to prevent unnecessary herbicide applications and also to prevent the adding of the current year’s herbicide resistant seeds to the seed bank. Here we propose a confirmed model to estimate *A*. *palmeri* population responses to trifloxysulfuron-methyl. This model is flexible as it can be adapted (after fitting modified parameters) to other troublesome weed species or crop tolerance to herbicides. The model can include safety as well as maintenance applications, that is, highway weed control and for removing weeds from fences as well as from parks and gardens. As the availability of hyperspectral sensors, computing power and machine learning techniques increases, we envisage that hyperspectral technologies determining resistance to a specific herbicide or herbicides will find ever-increasing application in weed control; for example, a sprayer with a hyperspectral ‘eye’ and a digital ‘brain’ would be able to deliver the most effective herbicide in real time. Such a system could also include non-destructive measurements for a variety of agricultural uses and the ability to collect seeds for future genetic studies.

## Conclusion

In a world in which crop resources are decreasing, input investments in agriculture are increasing, and technology (e.g., optical sensors) is becoming more readily available and cost effective, the proposed hyperspectral detection methods for herbicide response could have a significant impact on the optimal exploitation for agriculture of semi-arid areas and in other resource-poor environments. The current study may be regarded as a ‘feasibility check’ of an integrated model that can predict both ecological fitness of a field population (e.g., seed germination) and the response to a specific herbicide. The proposed system can be applied to prevent ineffective and unnecessary use of chemicals, thereby reducing costs and, more importantly, minimizing the overload of unnecessary chemicals in the environment. The proposed toolbox could also serve as a powerful tool for herbicide development by improving accuracy of dosages and timing and increasing the probability of early detection of responses to herbicides in weeds as well as in crops. This methodology can also be applied in weed-infested non-arable lands and for other weed species and other herbicides.

## Author Contributions

MM, IH, CN, TK, YZ, TI, DS, BR, AK, and HE all contributed to the current study and to writing the paper. MM, HE, and BR conceived and designed the study. CN constructed the seeds hyperspectral imaging system and analyzed the data. IH, AK, and TI designed methodology of leaf data collection. IH and MM obtained the leaf spectral measurements. YZ and MM obtained the leaf gas exchange measurements. IH analyzed the leaf spectral data. TK conducted and analyzed RGB images.

## Conflict of Interest Statement

The authors declare that the research was conducted in the absence of any commercial or financial relationships that could be construed as a potential conflict of interest.

## Footnotes

1 http://rsb.info.nih.gov/ij
