# Supplementary material for: Hyperspectral Technologies for Assessing Seed Germination and Trifloxysulfuron-methyl Response in Amaranthus palmeri (Palmer Amaranth)
Source: Front Plant Sci. 2017 Apr 3;8:474. doi: 10.3389/fpls.2017.00474 (PMC5376577; doi:10.3389/fpls.2017.00474)
Supplement: Supplementary file 2 [file Data_Sheet_1.PDF]

Supporting Information

Hyperspectral technologies for assessing seed germination and trifloxysulfurone-methyl response in *Amaranthus palmeri* (Palmer amaranth)

Maor Matzrafi[1§], Ittai Herrmann[2*§], Christian Nansen[3, 4], Tom Kliper[1], Yotam Zait[1], Timea Ignat[5], Dana Siso[6], Baruch Rubin[1], Arnon Karnieli[2] and Hanan Eizenberg[6]**

[1]*The Robert H. Smith Institute of Plant Sciences and Genetics in Agriculture, The Robert H. Smith Faculty of Agriculture, Food and Environment, The Hebrew University of Jerusalem, P.O. Box 12, Rehovot 7610001, Israel;* [2]*The Remote Sensing Laboratory, Blaustein Institutes for Desert Research, Ben-Gurion University of the Negev, Sede Boker Campus 8499000, Israel;* [3]*Department of Entomology and Nematology, University of California Davis, Davis, California 95616, USA;* [4] *State Key Laboratory Breeding Base for Zhejiang Sustainable Pest and Disease Control, Zhejiang Academy of Agricultural Sciences, 198 Shiqiao Road, Hangzhou 310021, China;* [5]*Institute of Agricultural Engineering, Volcani Center, Agricultural Research Organization, Bet Dagan 50250, Israel; and* [6]*Department of Plant Pathology and Weed Research, Agricultural Research Organization, Newe Ya'ar Research Center, Israel*

[§]These authors contributed equally to this work.

*****Current affiliation - Department of Forest & Wildlife Ecology, University of Wisconsin-Madison, Madison, WI 53706, USA.

**Corresponding author: eizenber@volcani.agri.gov.il

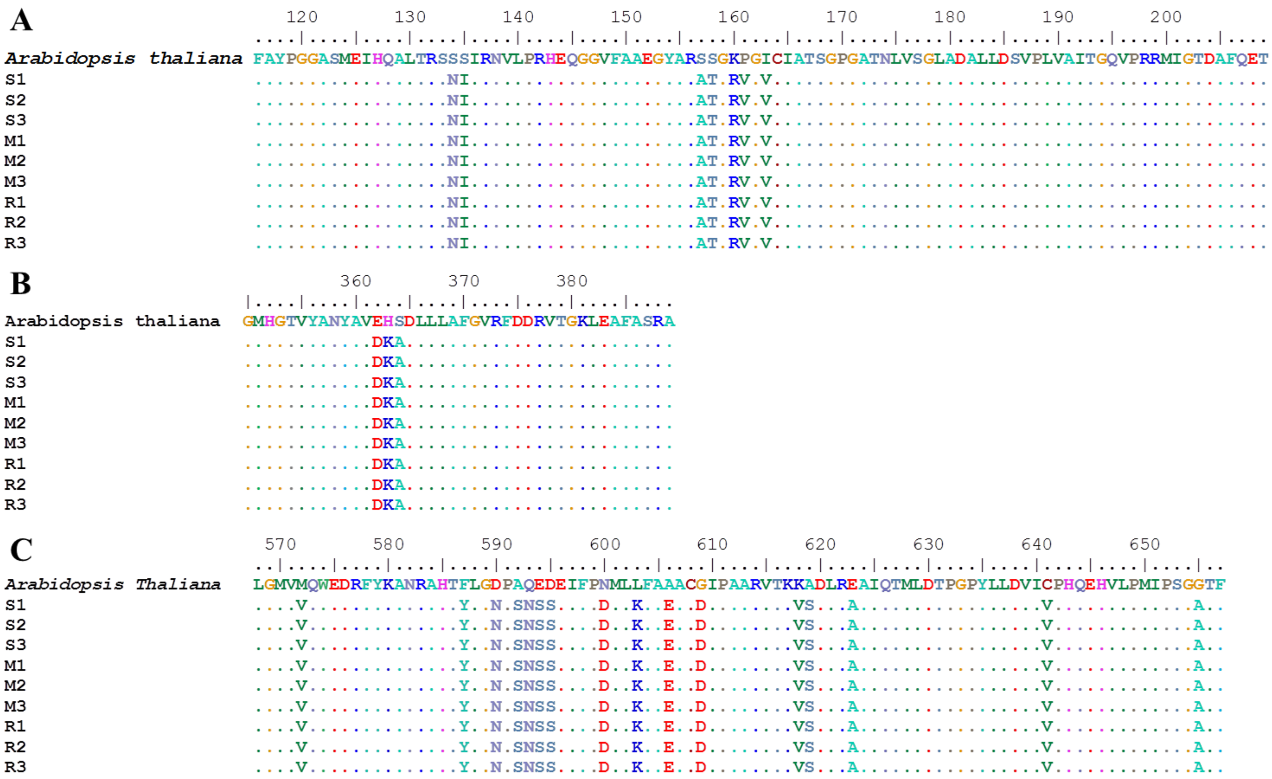

Figure S1. Alignment of partial *ALS* sequences of *A. palmeri* plants from different response groups: sensitive (S), moderate (M) and resistant (R). All three parts (**A**, **B** and **C**) of the gene were sequenced and positions refer to the *ALS* sequence of *A. thaliana* (X51514).

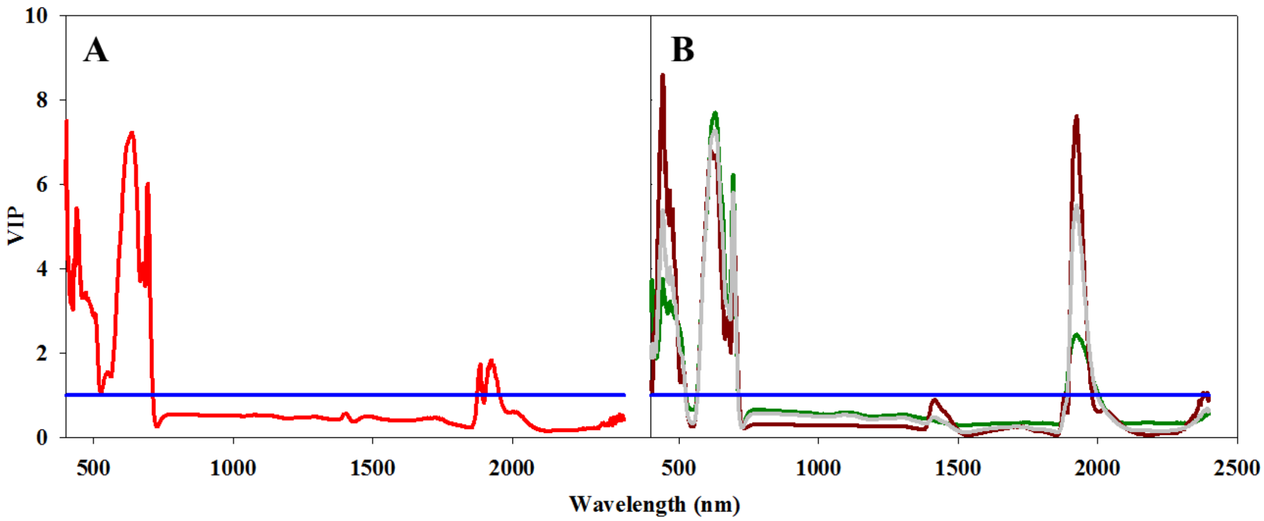

Figure S2. Variable Importance in Projection (VIP) models for different classes of a partial least squares–discriminant analysis (PLS-DA) classification model, 1 (blue line), is a putative threshold considered for influence on the model. (**A**) Two-class general VIP model and (**B**) three-class model, with different VIP for each of the response groups: moderate (brown), sensitive (gray) and resistant (green).
